# Annotations

**Published:** 1896-02-08

**Authors:** 


### ANNOTATIONS.

### More Dangers from Tuberculosis.

Now that it is shown that tuberculosis is a bacillary disease, it is easy to make a diagnosis with certainty in whatever part of the body its presence may be suspected, by demonstrating the presence of the active tubercle bacillus at the diseased locality. Tuberculosis, as is obvious, may be inoculated into any part of the body; and, being inoculated at a given point, may develop there, and give rise to general tuberculosis, with fatal results. Several cases, interesting to the general public as well as to the medical profession, are given in the current number of the *British Journal of Dermatology*. For example, Dr. White has recorded the case of a young girl whose tuberculosis first showed itself in the hand, and from the hand spread all over the body, finally killing the patient. The disease had been inoculated into the hand by the girl's washing of some pocket-handkerchiefs used by a consumptive patient. In passing, let me repeat the caution that consumptives should not be allowed to use pocket-handkerchiefs, but only prepared paper, and that the paper should be promptly burnt. A still more curious instance occurred in the case of a girl who was inoculated with the disease in the process of having her ears pierced. The friend who pierced the ears, and a sister who afterwards dressed the wounds, were both consumptive. All these dangers, so obvious to the experienced bacteriologist, are by no means yet understood by the public.

### The Lettsomian Lectures.

On Monday evening Mr. Watson Cheyne gave the first of the Lettsomian Lectures before the Medical Society of London, taking for his subject "The Objects and Limits of Operations for Cancer," and referring especially to cancer of the breast. Far too much has it been the custom of surgeons to regard operations for such cases as being meant merely to afford some relief and postponement of the end, and to consider the possibility of cure as a thing almost too good to hope for. But Mr. Watson Cheyne showed that by carrying to its logical conclusion the knowledge we possess as to the track along which cancer becomes diffused, and by removing all those tissues in which we know that the infection spreads, far better results can be obtained than have hitherto been looked for, 57 per cent. of the cases which he had operated upon between 1890 and February, 1893, that is, cases which have now been done over three years, having remained free from recurrence. There is no denying that this is a splendid record, or that it opens out to many sufferers a prospect which will do much to take away that utter hopelessness which is often the cruellest feature in the downward progress of this disease. It is also to be noted that these results have not been obtained by limiting operation to early periods, for many of these operations were done in cases in which, according to some authors, systemic infection must necessarily have already taken place. The secret lies in the completeness of the removal. All the skin covering the tumour, the whole sheet of cellular tissue in which the breast lies, the fascia of the pectoral muscle, the contents of the axilla right up to the axillary vessels, all must be removed in one piece. If the skin will come together, well and good; if not, skin-grafting must be done. But, whatever happens, the disease must either be removed in one piece or it must be let alone. Some authors have said that when the glands are already affected there is no hope of cure, but this is by no means the experience of Mr. Watson Cheyne, who attributes more importance to the activity of the infective process than to the extent to which the infection has proceeded. He is then by no means over strict in his selection of cases, his limit being, (a) that the cancer shall not be in the form spoken of as *en cuirasse*; (b) that the infiltration of the axilla shall not implicate the nerves passing to the arm; (c) that the glands above the clavicle shall not be obviously affected; and (d) that there shall be no evident secondary deposits. This leaves open a far wider field for operation than some would allow, and, after all that we have heard of late about the increased prevalence of cancer, it is a cause for much satisfaction to find that so much of it is capable of being permanently removed. The moral of all this is that a "lump in the breast," in patients over thirty-five, is not a case for watching but for immediate and thorough treatment, and that an operation is not a mere palliation, but may be undertaken in the full hope of cure.

### The Extension of Rabies.

Each case that occurs is derived *de novo* from a diseased animal, and thus in practice the prevention of the disease in man depends upon the means taken to prevent it from spreading among animals. It is therefore somewhat alarming to find the following remarkable increase in the reported cases of rabies recorded in the *London Gazette*. In 1892 there were 40 cases; in 1893, 94 cases; in 1894, 258 cases; in 1895, 723 cases. With this growing frequency in the occurrence of rabies it cannot be long before a corresponding increase takes place in the cases of hydrophobia. The localisation of the outbreaks, moreover, must not for a moment be looked upon as justifying merely local measures for their suppression. One peculiarity in rabid dogs is their tendency to wander and to snap; it is a form of delirium Trotting steadily but wearily along they travel through village after village, and although they appear to take but little notice of things around, they furiously attack anything that obstructs their path, and thus it happens that one wandering dog will often infect a whole series of places, sometimes biting men and children—giving rise to hydrophobia, more often worrying other dogs, and thus leaving behind it a trail of rabies. It is only by including large areas, then, that muzzling orders can be effectual in controlling a disease like this, and it becomes a very serious question whether the merely local orders which are so often imposed are worth the trouble and vexation which they always cause. What is wanted is a complete and all-embracing muzzling over the whole of the island, continued for such a time as to give reasonable prospect of exterminating the disease. We might then hope for some years, at least, to be at peace. We are all glad, however, as a small step in the right direction, to find that London has now joined with Middlesex and Surrey in their attempts to cope with the disease.

